# Fabrication of Fullerene Anchored Reduced Graphene Oxide Hybrids and Their Synergistic Reinforcement on the Flame Retardancy of Epoxy Resin

**DOI:** 10.1186/s11671-018-2678-z

**Published:** 2018-11-03

**Authors:** Rui Wang, Lixin Wu, Dongxian Zhuo, Zhengzhou Wang, Tsung Yen Tsai

**Affiliations:** 1 Fujian Institute of Research on the Structure of Matter, Chinese Academy of Sciences, Fuzhou, 350000 People’s Republic of China; 2 University of the Chinese Academy of Sciences, Beijing, 100049 People’s Republic of China; 3 Quanzhou Normal University, Quanzhou, 362000 People’s Republic of China; 4 School of Materials Science and Engineering, Tongji University, Shanghai, 201804 People’s Republic of China; 5 Department of Chemistry, Center for Nanotechnology, R&D Center for Membrane Technology, Chung Yuan Christian University, Chungli, 32023 Taiwan

**Keywords:** Graphene, Fullerene, Thermosetting resin, Flame retardancy, Mechanical property

## Abstract

A C_60_-PEI-rGO hybrid was prepared by incorporating the fullerene (C_60_) on the surface of PEI-modified reduced graphene oxide (rGO) and then used to modify the epoxy (EP) resin. Subsequently, the structure of GO and C_60_-PEI-rGO hybrid were well characterized, showing that the C_60_ was homogenously anchored on the surface of PEI-rGO. The flame retardancy, mechanical properties, and thermal stability of as-prepared C_60_-PEI-rGO/EP nanocomposites were systematically investigated. Results show that the C_60_-PEI-rGO hybrid exhibits high flame retarding efficiency for EP. Specifically, the time to ignition of epoxy increases from 68 to 89 s with the addition of 1.0 wt% C_60_-PEI-rGO, which are unusual in polymer nanocomposites. In the meantime, the peaks of the heat release rate and total heat release of the modified epoxy reduce by 40.0% and 15.6%, respectively. The synergistic flame retardant mechanism of C_60_-PEI-rGO to EP is attributed to its unique structure combining both the high efficiency in capturing free radicals by C_60_, the barrier effect of layered of rGO and increase of crosslinking density of epoxy. It is shown that the thermal stability and mechanical properties of epoxy are simultaneously improved with the addition of C_60_-PEI-rGO. This work may pioneer a new and efficient method to fabricate fire retardant thermosetting resins with simultaneously other improved properties.

**Electronic supplementary material:**

The online version of this article (10.1186/s11671-018-2678-z) contains supplementary material, which is available to authorized users.

## Background

Polymeric materials have been widely applied in many fields such as construction, electrics and electronics, and coating, due to their lightweight, processing capability, and versatile properties [1–3]. However, most of polymers are flammable which often causes the safety concern [4]. Therefore, improving the flame retardancy of polymeric materials has been tremendously demanded.

Comparing with traditional flame retardants, the nanofillers not only exhibit the higher flame retarding efficiency for polymeric materials but also keep the other original properties, even endow polymeric materials with some special functionalities at the low additions, which have attracted much attentions [5, 6]. Thereinto, the flame retarding effects of nanofillers mainly embody with the remarkable decrease of typical parameter such as peak heat release rate (PHRR), total heat release (THR), and total smoke release (TSR), or an increase of limiting oxygen index (LOI).

However, it is found that the flame retarding efficiency of nanofillers on the thermoplastics and thermosetting resins are different. With regard to thermoplastic resins, the nanofillers can show a significant improvement on flame retardancy. For example, Gilman et al. incorporated the surfactant modified montmorillonite into polypropylene graft maleic anhydride (PPgMA) by melt blending, and the resultant PPgMA-MMT (4 wt%) nanocomposite had a 75% lower PHRR and 49% lower THR than the pure PPgMA, respectively only at the addition of 4 wt%, indicating the significant improvement on flame retardancy [7]. Also, the similar remarkable improvements also were observed in poly(methylmethacrylate) (PMMA)/carbon nanotube and polypropylene (PP)/graphene nanocomposites [8–11] . These nanofillers also had been applied to modify thermosetting resins for improving their flame retardancy [12, 13].

However, it is noted that the flame retarding efficiencies of these nanofillers in thermosetting resins are not pronounced as theirs in thermoplastic resins. In our previous work, graphene oxide (GO) was incorporated to epoxy resin (EP), and the resultant nanocomposites only exhibited a decrease of 16% on the PHRR at the 1 wt% content of GO compare to that of EP [14]. Guo et al. showed the similar phenomenon in graphene/EP, which had a decrease of 23% on the PHRR of epoxy at the 1 wt% content of graphene compare to that of EP. The reasons behind this phenomenon can be explained by the effect of nanofillers on the crosslinking structure as well as the roles of nanofillers on combustion of polymer. On the one hand, due to the comparatively high crosslinking densities of thermosetting resins, the addition of nanofillers is difficult to significantly change the crosslinking density which plays a determinate role in improving the flame retardancy [15, 16]. On the other hand, the mechanism of nanofillers on flame retardancy of polymer is singular, which mainly depend on their barrier effect [17, 18] and then hard to exert high modified efficiency. Obviously, comparing with these attractive progresses in thermoplastics, the modified efficiencies of nanofillers in thermosetting resins needed to be further enhanced. Many efforts have been dedicated in modifying nanofillers with other flame retardants [12, 19]. For example, Hu and his coworkers modified graphene with octa-aminophenyl polyhedral oligomeric silsesquioxanes (OapPOSS) to obtain the OapPOSS-rGO, which exhibits remarkable flame retardant effect on epoxy resin [20]. However, some important index such as time to ignition (t_ign_) and time to peak of heat release rate are rarely reported, and the synergistic mechanism can be further studied.

Recently, fullerene (C_60_) had been incorporated into polymer for improving the flame retardancy of polymer due to its high reactivity towards free radicals which can act as a radical trapping reagent to delay the thermo-oxidative degradation of polymer [21–24]. However, C_60_ nanoparticles tend to agglomeration in polymer due to its large specific surface area and strong Van de Waals, which leads to the low flame retardant efficiency. Because of the same chemical composition, C_60_ was synergy with other carbon nanofillers, it not only improves the dispersion of nanofillers but also combines the radical absorption of C_60_ and the flame retardant effect of other carbon nanofillers such as the barrier effect of graphene, which further enhances the flame retardant efficiency of C_60_ [25–27]. Comparing with one dimension carbon nanomaterials such as carbon nanotube, graphene shows higher barrier efficiency due to its layered nanostructure and provides a more active platform to synergy with C_60_ [28]. Therefore, it would be interesting if these flame retarding mechanisms of C_60_ and graphene can be synergistically applied into polymer. Fang and his coworkers combined GO and C_60_ to obtain nanohybrid, and it largely improved the flame retardancy and thermal stability of HDPE [29]. However, to date, all these C_60_-related nanomaterials were incorporated into thermoplastic resins, while no researcher investigates their flame retardant effect and mechanism in thermosetting resins.

Herein, we designed a graphene-related hybrid (C_60_-PEI-rGO) consisting of graphene and fullerene (C_60_) through a three-step reaction, and incorporated into epoxy resin. The loose lamellar and amino-rich structure of C_60_-PEI-rGO may not only achieve the ideal dispersion of graphene and C_60_ in epoxy (EP) which will fully exert the radical absorption of C_60_, barrier effect of graphene, and increase the crosslinking densities of the resultant nanocomposites, but also may improve other typical properties of the resultant nanocomposites. It is believed that this work may pioneer a new and efficient method to fabricate fire retardant thermosetting resins with simultaneously other improved properties.

## Methods

### Materials

Graphite (3000 mesh) was supplied by Aladdin Industrial Co. Ltd. (China). Sulfuric acid (H_2_SO_4_, 98%), sodium nitrate (NaNO_3_), potassium permanganate (KMnO_4_), hydrogen peroxide (H_2_O_2_, 30% aq.), ethanol, dimethyl sulfoxide (DMSO), toluene, and acetone were commercial product with analytical grades and used without further purification. Distilled water was produced in our lab. C_60_ (purity > 99%) was bought from Henan Puyang Co. Ltd. Branched polyethlyamine (PEI, 50% aq.) was purchased from Sigma-Adrich with Mn of of 70,000. Diglycidyl ether of bisphenol A (DGEBA) was purchased from Shanghai Resin Factory Co. Ltd. (China). The curing agent diethyltoluenediamine (DETDA) was obtained from the Chongshun Chemical Co. Ltd. (China).

### Preparation of C_60_-PEI-rGO

**Scheme 1:**
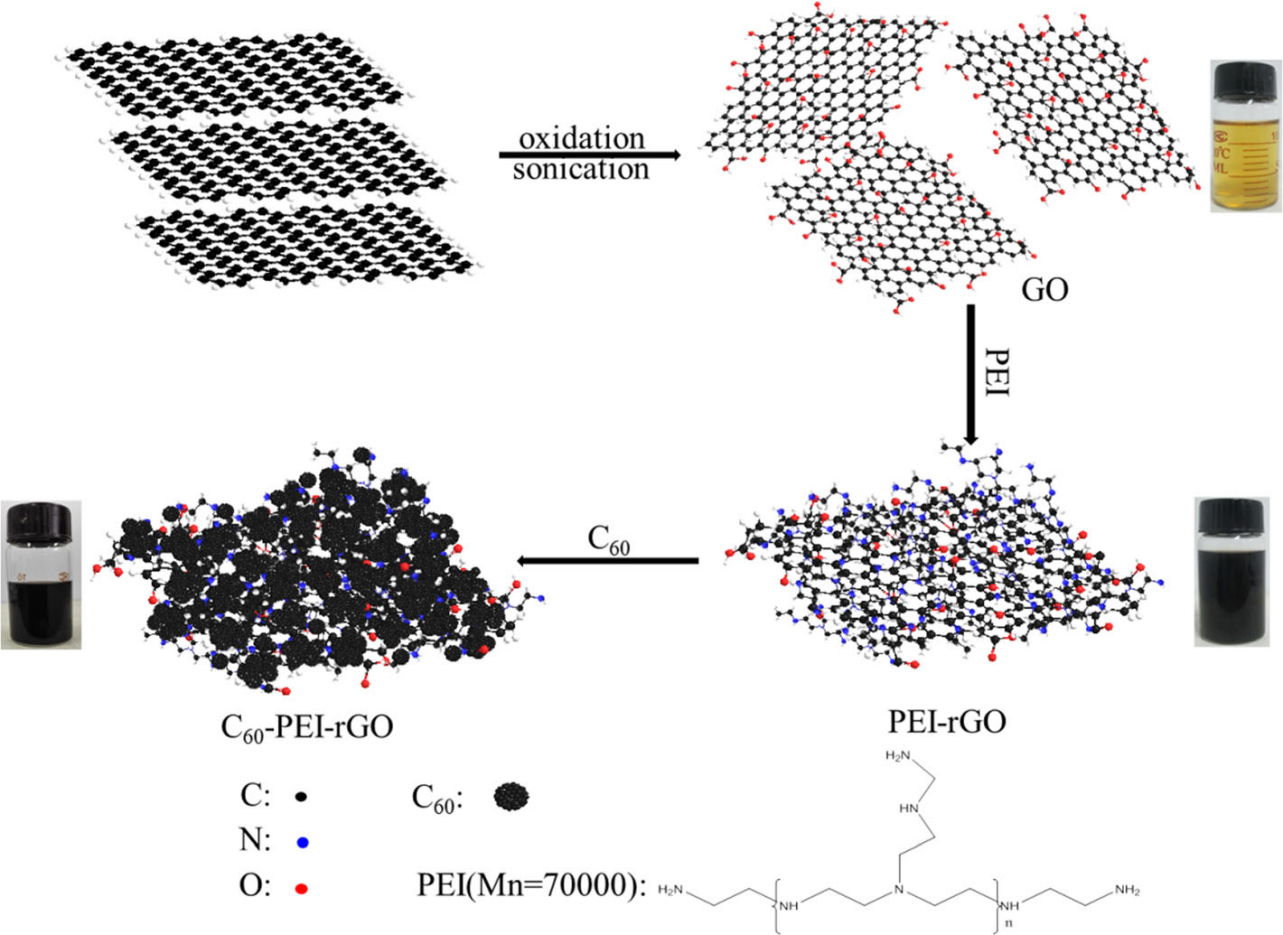
Schematic illustration of the preparation of C_60_-PEI-rGO

Graphite oxide (GO) was prepared using a modified Hummer’s method from graphite powders as shown in the Additional file 1 [30, 31]. PEI-modified reduced graphene oxide (PEI-rGO) was prepared by the reaction between PEI and graphene oxide as shown in the Additional file 1. After that, PEI-rGO (150 mg) was dispersed in DMSO (300 mL) by ultrasonication for 30 min. The PEI-rGO/DMSO solution and 300 mg of C_60_ were added into the DMSO-toluene (350 mL, 4:3, *v*/*v*) solution by ultrasonication; then, the resultant mixture was stirred at 90 °C for 24 h after ultrasonication for 30 min at room temperature. Finally, the product was washed with toluene and ethanol sequentially at least three times followed by drying at 60 °C under vacuum for 12 h, designated as C60-PEI-rGO. The preparation process of C60-PEI-rGO is shown in Scheme 1.

### Preparation of EP Resin and Nanocomposites

Appropriate amounts of DGEBA and DETDA with a weight ratio of 1:0.234 were blended at 100 °C for 15 min with vigorous stirring to obtain a light yellow liquid, which was EP prepolymer. And then, the mixture was thoroughly degassed in a vacuum oven at 110 °C for 30 min, followed by pouring it into a pre-heated (100 °C) “U”-type mold. Subsequently, the mold was put into an oven for curing and postcuring following the protocol of 120 °C/1 h + 180 °C/2.5 h and 190 °C/2 h, respectively; the resultant resin is a cured EP resin. Appropriate amounts of PEI-rGO, C60, and C60-PEI-rGO were respectively added into the mixture of EP prepolymer and ethanol by sonication for 30 min to form a black suspension, and then, the mixture was degassed to remove excess solvent at 60 °C in a vacuum oven. After that, the mixture was cast into a mold for curing and postcuring via the procedures of 120 °C/1 h + 180 °C/2.5 h and 190 °C/2 h, respectively. Finally, the resultant nanocomposites were demolded and coded as PEI-rGO1.0/EP, C601.0/EP C60-PEI-rGO*n*/EP, respectively, where 1.0 and *n* represent the weigh percent of used nanomaterial in the resultant nanocomposites (*n* = 0.4, 0.6, 0.8, and 1.0).

### Apparatus and Experimental Method

The morphology and microstructure of as-prepared nanomaterials and nanocomposites were characterized by an Atomic Force Microscope (AFM) (Veeco Instruments, Nanoscope Multimode IIIa, USA), a Transmission Electron Microscopy (TEM) (JEOL JEM-2010, Japan), a Scanning Electron Microscope (SEM) (HITACHI, SU8010/EDX, Japan), a Fourier Transform Infrared (FTIR) spectrometer (AVATAR360N, USA). Thermogravimetric analyses (TGA) of nanofillers were performed on a TA Instruments STA449C (USA) in the range from 25 to 800 °C under a nitrogen atmosphere with a heating rate of 10 °C/min, and epoxy and its composites were performed in the range from 25 to 800 °C under an air atmosphere with a heating rate of 10, 20, 30, and 40 °C/min. Dynamic mechanical analysis (DMA) was performed using TA DMA Q800 apparatus from TA Instruments (USA) from 25 to 250 °C with a heating rate of 3 °C/min at 1 Hz. The tensile properties were performed according to ASTM D638 with a constant speed of 5 mm/min using a load cell of 1 kN. LOI values were measured on a Stanton Redcraft Flame Meter (China) according to ASTM D2863/77. Flammability of the resins was characterized using a cone calorimeter performed in an FTT device (UK) according to ISO 5660 with an incident flux of 35 kW/m^2^ using a cone shape heater.

## Results and Discussion

### Characterization of GO, PEI-GO, and C_60_-PEI-rGO

In general, GO is difficult to disperse well in commonly used organic solvents [32]. However, in the preparation of PEI-rGO and C_60_-PEI-rGO, it is noteworthy that as-prepared PEI-rGO and C_60_-PEI-rGO can readily disperse in ethanol and formed the stable colloidal suspension, which can be attributed to the high compatibility between PEI and ethanol. The result provides the favorable condition for the exfoliation and dispersion of GO in the preparation of graphene-based nanocomposites. Moreover, there is a color transformation from yellow GO in water to black PEI-rGO and C_60_-PEI-rGO in ethanol, reflecting that the reduction of GO occurs.

**Fig. 1:**
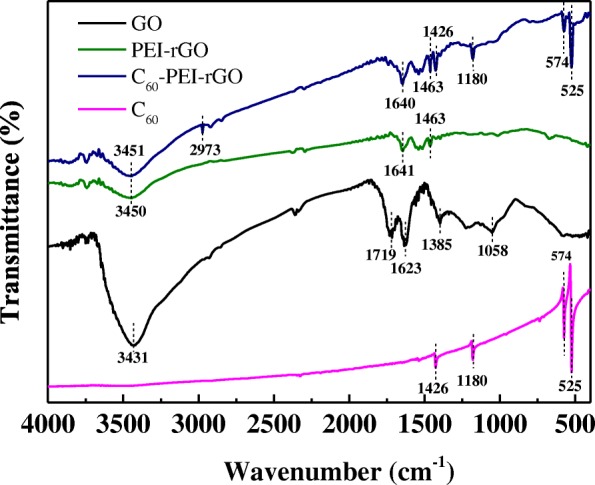
FTIR spectra of GO, PEI-rGO, C_60_-PEI-rGO, and C_60_

Figure 1 shows the FTIR spectrum of GO, PEI-rGO, C_60_-PEI-rGO, and C_60_. After complexation with PEI, the intensity of H-bond peak at 3431 cm^−1^ obviously decreases due to the partial reduction of GO by the PEI molecules, and the characteristic band at 1719 cm^−1^ completely disappears, along with obvious weakening of two peaks at 1385 (O–H) and 1058 (C–O) cm^−1^. The strong band at 1623 cm^−1^ in GO is pertinent to the skeletal vibration of un-oxidized graphitic domains, which is replaced by a strong band at 1640 cm^−1^ that is related to the formation of amide bonds [33, 34]. In addition, a new band at 1463 cm^−1^ (C–N stretching vibration) appears in PEI-GO due to the coverage of PEI to GO. For C_60_-PEI-rGO, four characteristic absorption peaks of C_60_ at 1426, 1180, 574, and 525 cm^−1^ and a new peak at 2973 cm^−1^ (C_60_–H) reflect the reaction between C_60_ and PEI-rGO, since un-reacted C_60_ were completely removed by washing the hybrid with toluene several times until the color of washed solution from aubergine to transparent under sonication [26].

**Fig. 2:**
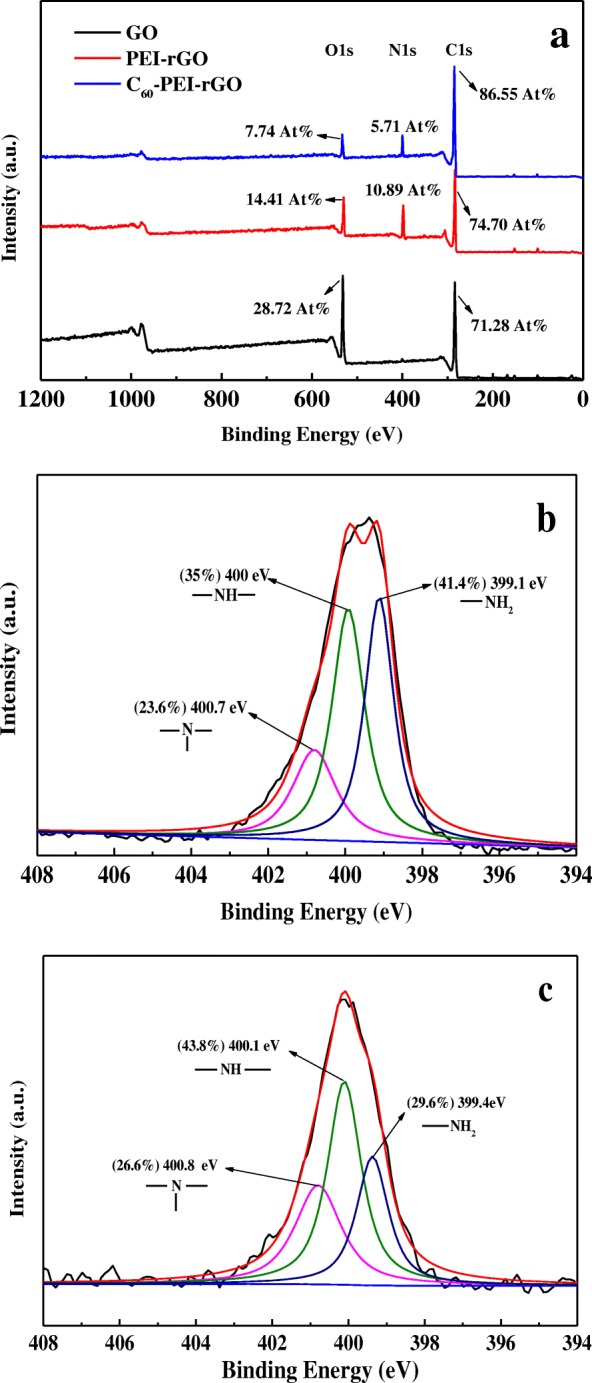
XPS spectra of GO, PEI-rGO and C_60_-PEI-rGO (**a**), and the N1s XPS spectrum of PEI-rGO (**b**), and C_60_-PEI-rGO (**c**)

Figure 2 shows the XPS spectra of the GO, PEI-rGO, and C_60_-PEI-rGO. It can be seen that two sharp peaks at 286.7 and 532.6 eV are corresponding to C and O elements in GO and its hybrids, respectively. The new sharp peaks at 399.7 eV (PEI-rGO) and 400.1 eV (C_60_-PEI-rGO) which correspond to N1s indicate the formation of amide bonds after functionalization. The rough chemical composition of GO and its hybrids are also showed in Fig 2a. For PEI-rGO, the content of O decrease from 28.72 to 14.41 at.% in GO, which is attributed to the introduction of PEI. In case of C_60_-PEI-rGO, content of O and N decrease to 7.74 at.% and 5.71 at.%, respectively, while the content of C reaches 86.55 at.%, which is an obvious evidence for C_60_ is introduced to PEI-rGO. It could roughly calculate that the weight ratio of C_60_ in C_60_-PEI-rGO is ca. 45.4 wt% according to “Mixture Rule” (at.% has been change to wt% in calculation). In N1s spectrum of the PEI-rGO (Fig. 2b) and C_60_-PEI-rGO Fig. 2c), the N1s spectrum of PEI-rGO is fitted to three peaks at ca. 399.1 eV (accounting for 41.4%), 400 eV (accounting for 35%), and 400.7 eV (accounting for 23.6%), assigning to the primary amines, secondary amines, and tertiary amines, respectively. For C_60_-PEI-rGO, the N content in tertiary amines and in secondary amines increase to 26.6% and 43.8%, respectively, while the N content in primary amines significantly decreases to 29.6%. Based on the increase of the N content in secondary amines and the decrease of the N content in primary amines, it shows that C_60_ mainly reacts with primary amines to produce secondary amines, and a small amount of C_60_ react with secondary amines to produce tertiary amines, which can be attributed to the steric effect of C_60_ and the chemical activity of amines.

**Fig. 3:**
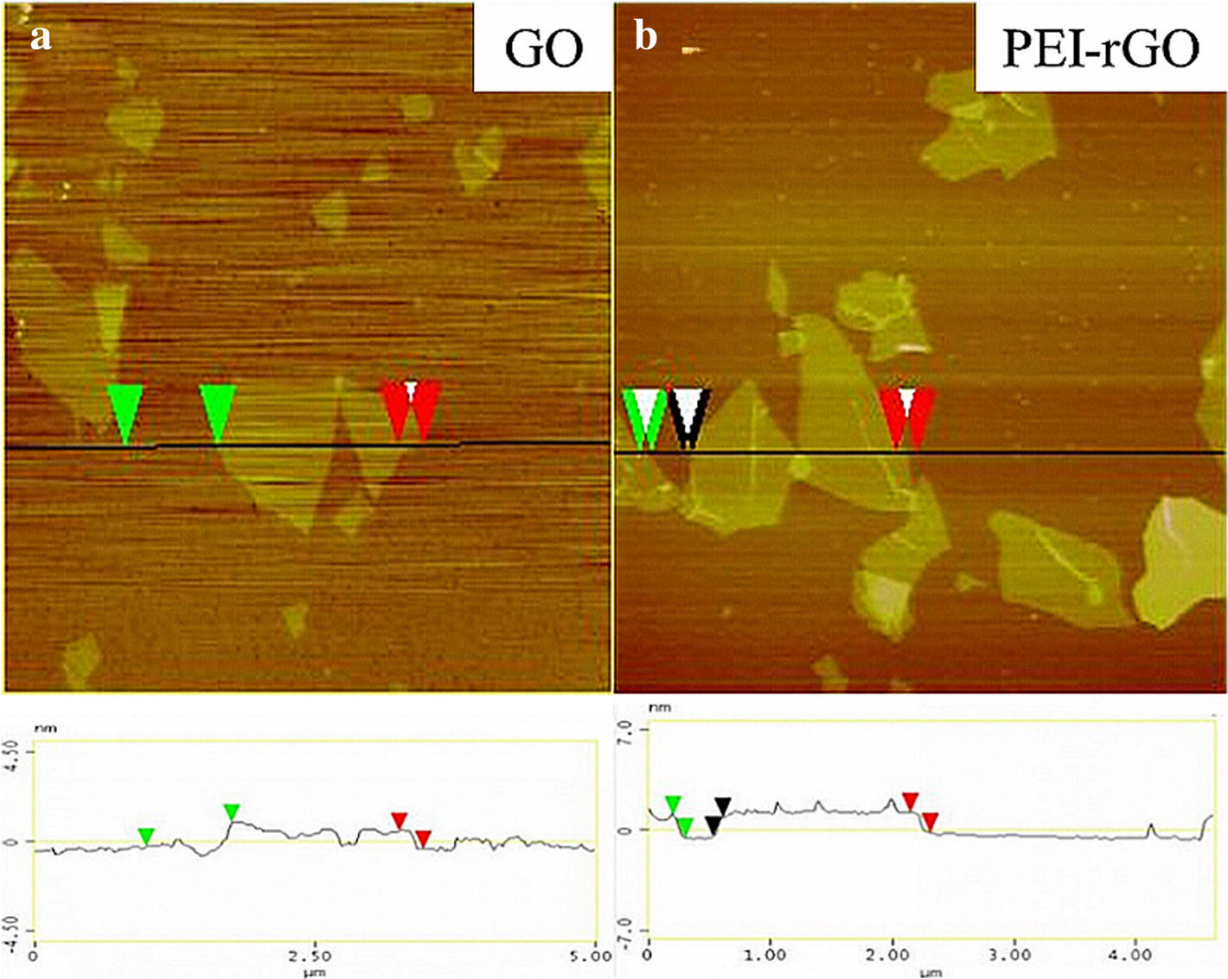
AFM images of GO (**a**) and PEI-rGO (**b**)

**Fig. 4:**
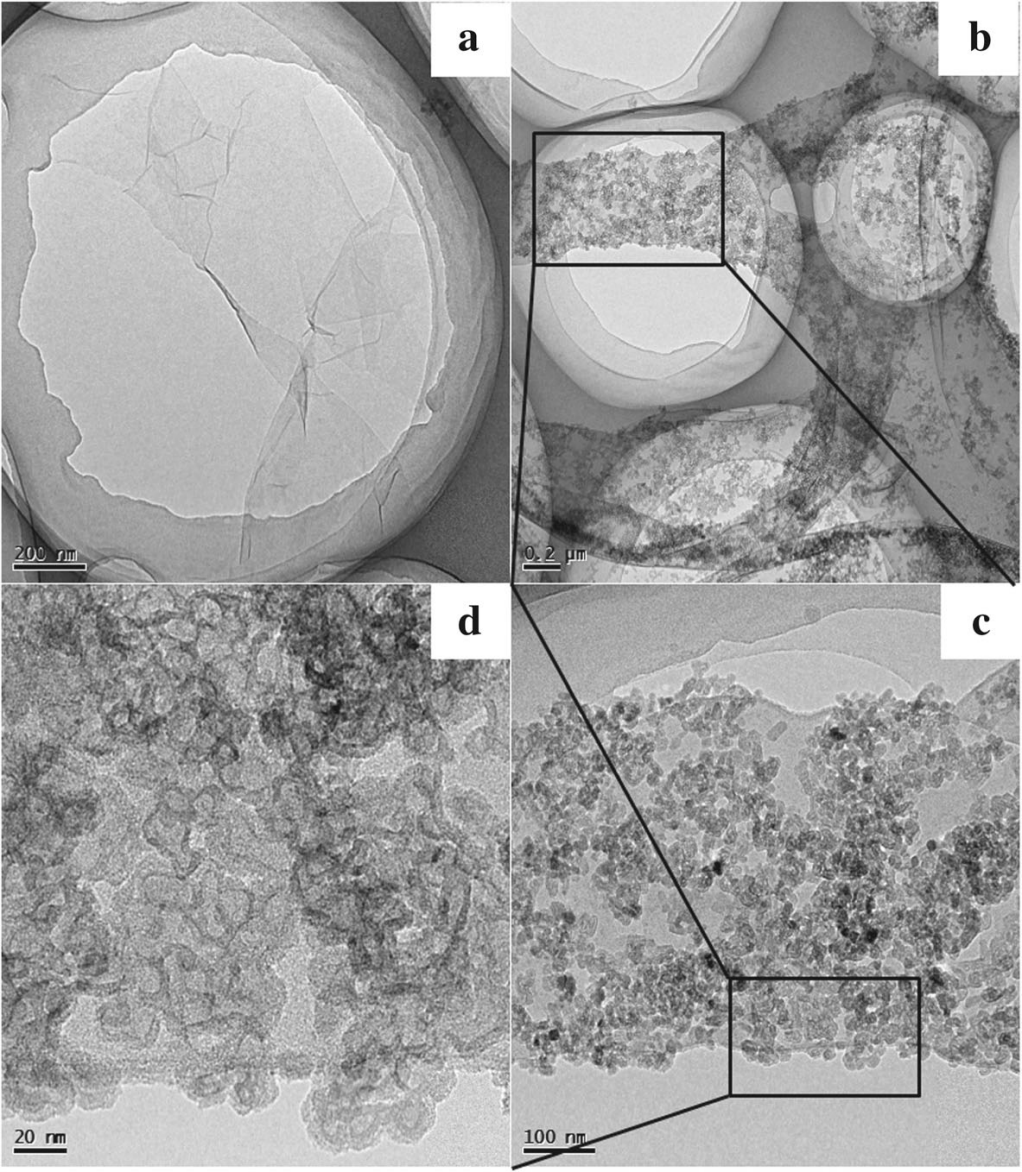
TEM images of PEI-rGO (**a**) and C60-PEI-rGO (**b**, **c**, **d**)

The morphologies of GO, PEI-rGO, and C_60_-PEI-rGO were investigated by AFM and TEM. As shown in Fig. 3, the size of GO covers 0.2–1 μm and the thickness is ca. 0.9 nm, which indicates GO had been exfoliated and likely to be in form of single layer nanosheets. After the coverage of PEI, the thickness of the PEI-rGO nanosheet increases to ca. 1.5 nm with uniform surface height distribution. PEI molecules are absorbed on both sides of a GO sheet, that is, an average thickness ca. 0.3 nm. As shown in Fig. 4, PEI-rGO nanosheet exhibits a typically smooth layered structure, while it is interesting to find that the C_60_-PEI-rGO has a loose lamellar structure and ca. 20 nm C_60_ aggregations are uniformly distributed on the surface of PEI-rGO. It can be expected that this loose lamellar structure not only prevents the re-stacking of hybrid nanosheets during the drying process and leads to the uniform dispersion of hybrid nanosheets in polymer, but also improves the physical interaction between C_60_-PEI-rGO and EP matrix.

**Fig. 5:**
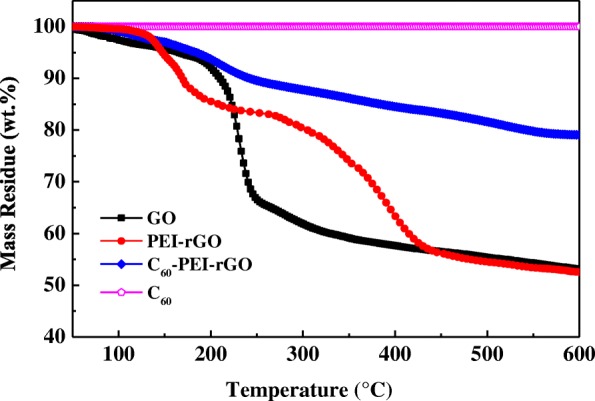
TG curves of GO, PEI-rGO, EP, C_60_-PEI-rGO, and C_60_ in a nitrogen atmosphere

The TGA curves of GO and its hybrids are shown in Fig. 5. The curve of GO shows ca. 4 wt% of weight loss below 150 °C due to the desorption of the absorbed water, and ca. 42.9 wt% of weight loss from 200 to 600 °C which can be attributed to decomposition of oxygen-containing groups. In the case of PEI-rGO, it can be seen that the weight loss ca. 15.6 wt% occurs from 100 to 190 °C which due to the decomposition of absorbed water, and the weight loss stage in 270 to 470 °C (ca. 26.7 wt%) which primary originates from the decomposition of PEI and more stable oxygen-containing groups. For C_60_, it shows high thermal stability in nitrogen with a mass residue of 99.3% at 600 °C. With regard to C_60_-PEI-rGO, the degradation rate obviously decreases, and the mass residue at 600 °C increases to 79.4%, which shows the highest thermal stability among GO and hybrids. By comparing the mass residue of PEI-rGO, C_60_, and C_60_-PEI-rGO, the weight ratio of C_60_ in C_60_-PEI-rGO could be calculated, ca. 55.2 wt%; this result has 10 wt% difference with the XPS result (45.4 wt%), but still can consider the weight ratio of C_60_ in C_60_-PEI-rGO is ca. 50 wt%.

Based on the above results, C_60_-PEI-rGO exhibits high compatibility with ethanol, leading to better dispersion in EP matrix than C_60_, or GO alone in EP is successfully prepared by chemically bonding PEI and C_60_, and it will finally affect the performance of the resultant nanocomposites.

### The Structure of C_60_-PEI-rGO/EP Nanocomposites

In general, the structure determines the performance of a material. Therefore, it is necessary to clarify the structure of the nanocomposite which involves the macro-structure such as the dispersion of nanofillers and micro-structure such as the interfacial interactions and cross-linking density of matrix.

**Fig. 6:**
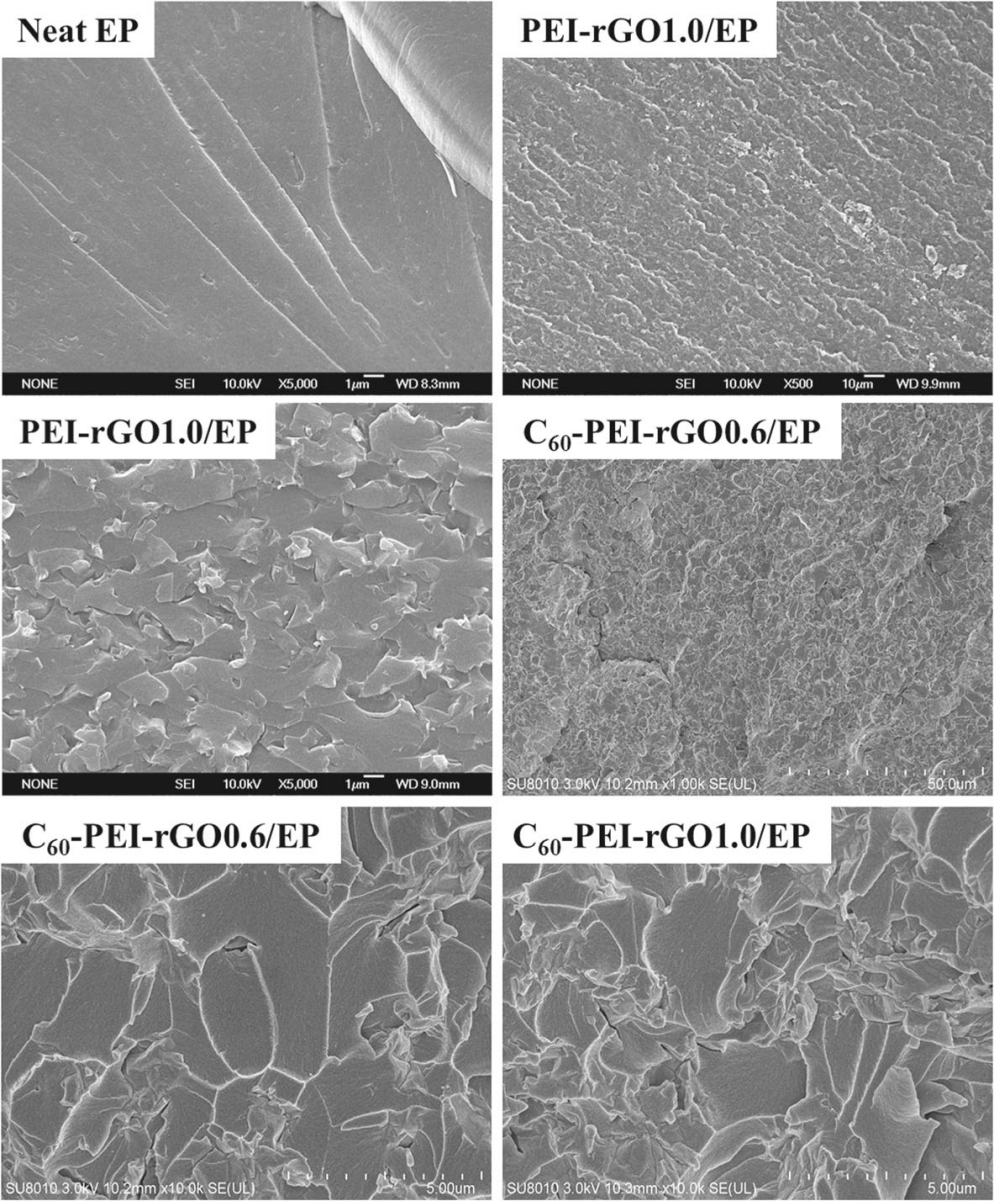
SEM images of cured EP resin and its nanocomposites

Functionalizing GO with the group is a wildly used approach to increase the interfacial interactions between the GO or graphene and polymer [35]. Herein, the amine groups in PEI molecules are designed to be introduced on the surface of GO, providing a guarantee for outstanding flame retardancy and integrated properties. Neat EP, PEI-rGO1.0/EP, and C_60_-PEI-rGO0.6/EP can be observed by SEM images of their fractured surfaces, as shown in Fig. 6. It can be seen that both PEI-rGO and C_60_-PEI-rGO are well dispersed in the EP matrix without any significant aggregation, and show high roughness, indicating the good dispersion of PEI-rGO and C_60_-PEI-rGO and strong interfacial interactions with EP.

**Fig. 7:**
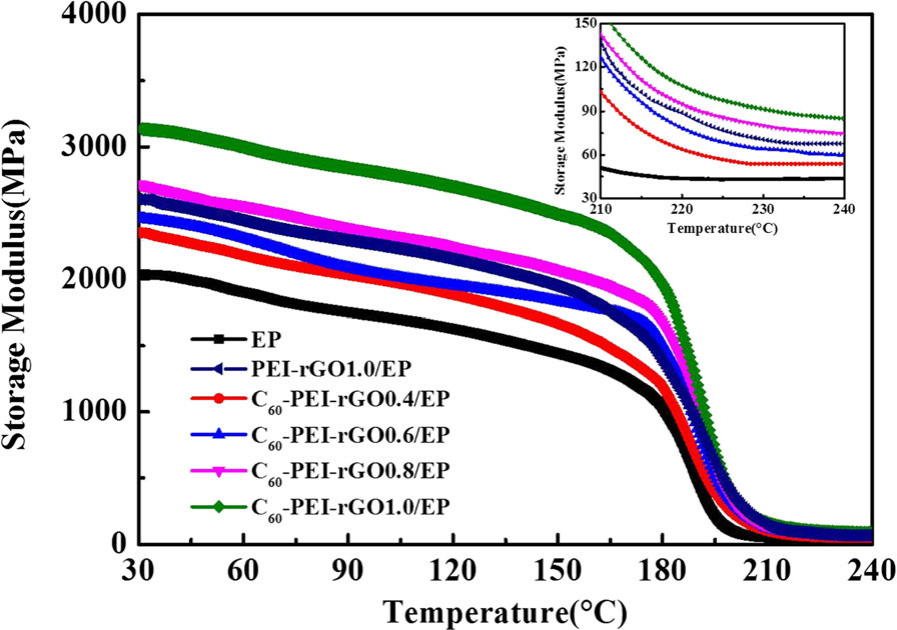
DMA curves of storage modulus (E′) of cured EP resin and its nanocomposites

The modulus of the rubber plateau, a parameter for polymer networks, is general indication of the degree of interaction between the polymeric system and the fillers [36]. As Fig. 7 shows, the C_60_-PEI-rGO/EP and PEI-rGO1.0/EP nanocomposite all exhibit higher modulus of the rubber plateau compare to that of neat EP, indicating the strong interfacial interaction between EP and hybrids. It is noted that the modulus of the rubber plateau of C_60_-PEI-rGO1.0/EP nanocomposite is higher than that of PEI-rGO1.0/EP. It can be explained that the amine groups of PEI bond with EP during cure process and act as the coupling points, which increase the cross-linking densities of nanocomposites, and besides that, for C_60_-PEI-rGO, it not only possesses the amine groups on the surfaces of GO but also has the rough surfaces which have stronger physical interaction with EP as discussed above. Generally, the addition of filler usually causes the loose stacking of the polymer chains and finally leads to weak interfacial interactions between fillers and polymer [37]. However, in this work, the amine groups in PEI-rGO and C_60_-PEI-rGO tend to shorten the distance among cross-linking points which result in the increase of cross-linking density of PEI-rGO1.0/EP and C_60_-PEI-rGO/EP, Moreover, the rough surfaces of C_60_-PEI-rGO can enhance the interfacial interactions between C_60_-PEI-rGO and EP by the physical interactions.

### Flame Retardancy and Mechanism of Cured C_60_-PEI-rGO/EP Nanocomposites

**Fig. 8:**
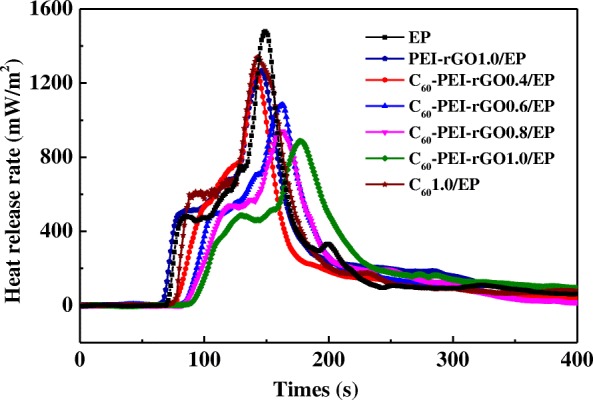
Dependence of heat release rate on time of cured EP resin and its nanocomposites

Cone calorimetry and LOI are two effective methods to evaluate the flame retardancy of polymeric materials. Figure 8 shows the curves from cone calorimeter for cured EP and its nanocomposites, and the typical parameters and LOI values are summarized in Table 1. It can be seen that the incorporation of C_60_-PEI-rGO to EP resin can significantly slow down the combustion process. Specifically, the t_ign_ and times to PHRR of C_60_-PEI-rGO/EP significantly increase. Especially, 21-s increment of t_ign_ and 28-s increment of times to PHRR occur in C_60_-PEI-rGO1.0/EP nanocomposite compare to those of neat EP, respectively. Meanwhile, C_60_-PEI-rGO/EP nanocomposites exhibit the remarkable lower PHRR and the slight lower THR compare to those of neat EP. Thereinto, C_60_-PEI-rGO1.0/EP displays 40.0% and 15.6% reduction in the PHRR and THR, respectively, comparing to those of neat EP. In addition, the LOI value of epoxy resin increases with the addition of C_60_-PEI-rGO, specifically, the cured C_60_-PEI-rGO0.8/EP nanocomposite has the maximum LOI value, 30.1%, which is about 1.18 times that of neat EP resin. Moreover, the LOI value of PEI-rGO 1.0/EP and C_60_1.0/EP are 27.5 and 26.3, respectively, which are lower than those of C_60_-PEI-rGO1.0/EP. Obviously, the C_60_-PEI-rGO exhibits high flame retarding efficiency for EP. In addition, it is noticeable that C_60_-PEI-rGO1.0/EP has a better flame retardancy than those of PEI-rGO1.0/EP and C_60_1.0/EP, further demonstrating that a remarkable synergetic effect between the functionalized GO and C_60_ on the enhanced flame retardancy can be exerted through covalent functionalizing of C_60_ on the surface of GO by PEI. As described above, the incorporation of C_60_-PEI-rGO into EP resin increases the crosslinking density, which is an important factor that leads to the improved flame retardancy of C_60_-PEI-rGO/EP nanocomposites. UL-94 vertical burning results of samples are given in Additional file 1: Table S1. The flame propagation speed is slightly decreased with the addition of C_60_-PEI-rGO. However, no samples can achieve a V-1 or V-0.

**Table 1:** Selected cone calorimeter data for pure EP and its nanocomposites

Samples	*t*_ign_(s)	PHRR (kW/m^2^)	PHRR reduction (%)	THR (MJ/m^2^)	Time to PHRR(s)	LOI (%)
EP	68	1479	–	101.9	149	25.5
PEI-rGO1.0/EP	65	1268	14.27	97.8	147	27.5
C_60_1.0/EP	72	1341	9.33	100.5	143	26.3
C_60_-PEI-rGO0.4/EP	76	1270	14.13	95.1	141	28.6
C_60_-PEI-rGO0.6/EP	82	1085	26.63	91.3	163	29.2
C_60_-PEI-rGO0.8/EP	84	937	36.65	90.2	163	30.1
C_60_-PEI-rGO1.0/EP	89	887	40.03	85.8	177	29.8

In order to further confirm the effect of C_60_-PEI-rGO on the flame retardancy, the thermal-oxidation stability of cured C_60_-PEI-rGO/EP and EP resins were evaluated because the flame retardancy of a polymer is directly related to whether the thermal-oxidative degradation step proceeds easily or not. In detail, thermal degradation kinetics of original and modified EP resins were calculated and compared by Kissinger’s method [38]. The thermo-gravimetric kinetics of a material can be calculated by Eq. 1:

1 $$ \ln \left(\beta /{T}^2\right)=\left(-{E}_{\mathrm{a}}/\mathrm{R}\right)\left(1/\mathrm{T}\right)-\ln \left[ ARn\left(1-\alpha \right)n-1/{E}_{\mathrm{a}}\right] $$

where *β* is the heating rate at the maximum degradation rate (*K*/min), *T* is the temperature at the maximum degradation rate (*K*), *E*_a_ is the activation energy (J/mol), *R* is the molar gas constant (= 8.314 J/mol K), *A* is the pre-exponential factor (1/s), *n* is the decomposition order, and *α* is the fraction of decomposition.

**Fig. 9:**
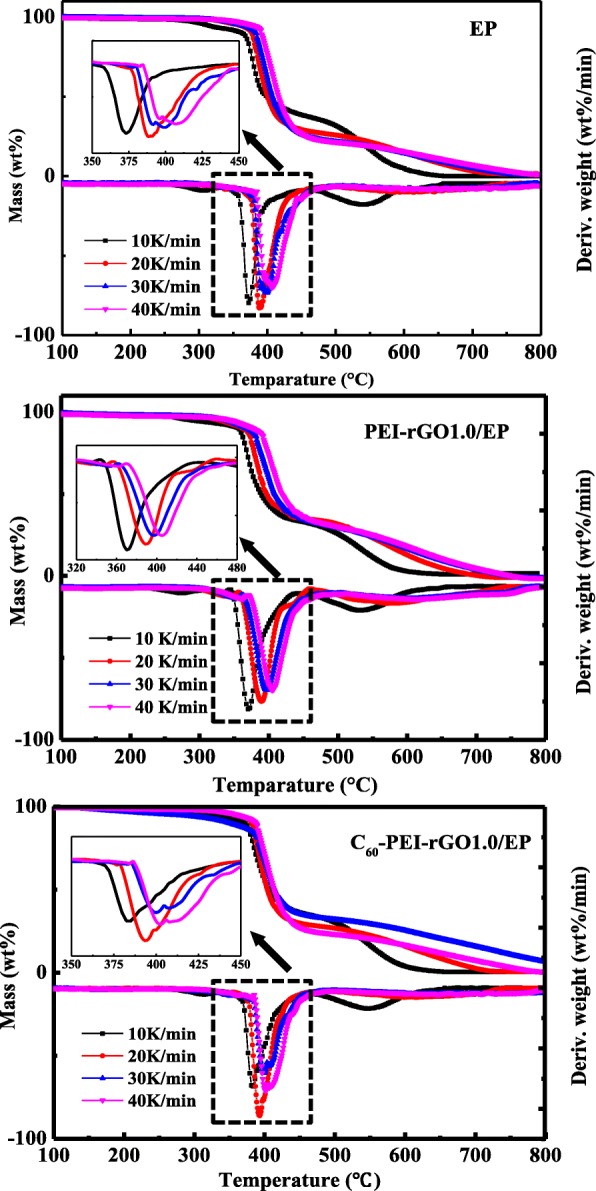
TG and DTG curves of cured EP resin, PEI-rGO1.0/EP nanocomposite, and C_60_-PEI-rGO1.0/EP nanocomposite in an air atmosphere with different heating rates

**Fig. 10:**
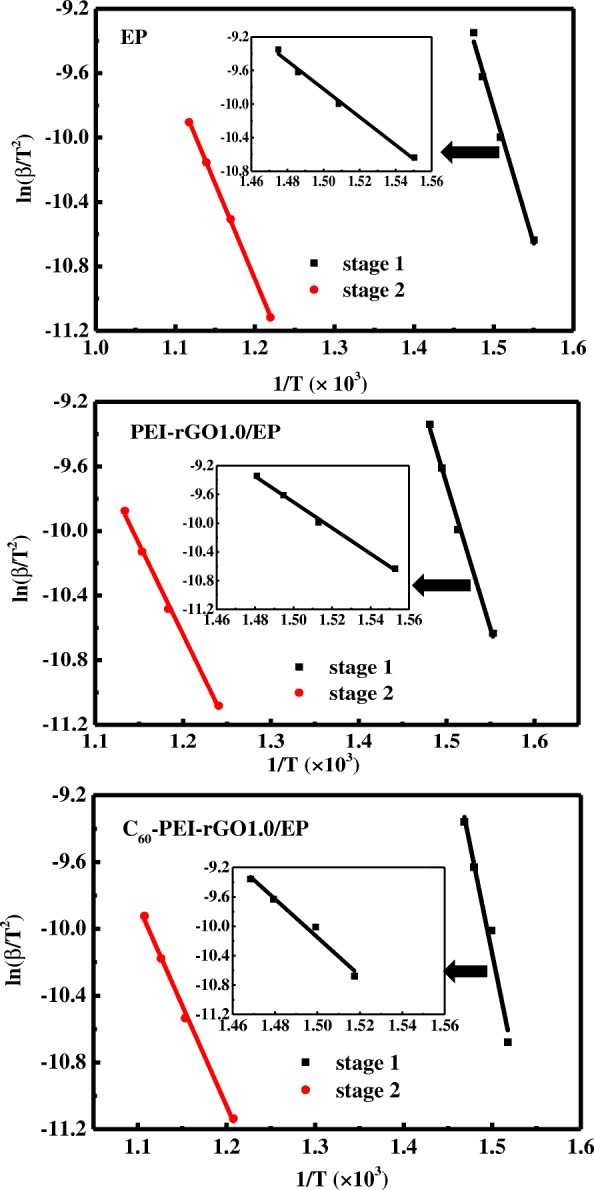
Plots of ln(*β*/*T*^2^) against 1/*T* for different decomposition stages of cured EP resin, PEI-rGO1.0/EP nanocomposite and C_60_-PEI-rGO1.0/EP nanocomposite

Four kinds of heating rates (10, 20, 30, and 40 K/min) were introduced to study the thermal degradation kinetics. Figure 9 shows TG and DTG curves of cured EP and its nanocomposites. Here, stage 1 and stage 2 are related to the decomposition of the macromolecular chains, and the oxidation of char residue, respectively. The relevant data from TG analyses of cured EP and cured EP nanocomposites at different heating rates in an air atmosphere are shown in Table 2. The activation energy (E_a_) can be obtained from the slope (−E_a_/R) of ln(*β*/*T*^2^) vs. 1/*T* plot (Fig. 10), and the calculated data are summarized in Table 3. The addition of C_60_-PEI-rGO to EP resin significantly changes the value of E_a_ at 1st degradation stage to varying degrees, and the increments increase when continuing to increase the loading of C_60_-PEI-rGO. However, the value of *E*_a_ is while slight varies at 2nd stage.

**Table 2:** Characteristic data from TG analyses of cured EP and its nanocomposites in an air atmosphere

	*β* = 10 K/min		β = 20 K/min		β = 30 K/min		β = 40 K/min	
Sample	*T*_max1_ (°C)	*T*_max2_ (°C)	*T*_max1_ (°C)	*T*_max2_ (°C)	*T*_max1_ (°C)	*T*_max2_ (°C)	*T*_max1_ (°C)	*T*_max2_ (°C)
EP	372	547	390	587	400	605	405	622
PEI-rGO1.0/EP	371	533	388	572	395	594	402	609
C_60_1.0/EP	374	564	389	598	398	617	405	634
C_60_-PEI-rGO0.4/EP	372	548	389	586	396	604	402	625
C_60_-PEI-rGO0.6/EP	380	560	393	601	400	619	405	631
C_60_-PEI-rGO0.8/EP	386	555	394	594	403	615	408	630
C_60_-PEI-rGO1.0/EP	388	560	394	597	403	616	409	633

**Table 3:** Thermal-oxidative decomposition kinetics parameters of cured EP and its nanocomposites

Sample	Region	Activation energy E_a_ (KJ/mol)	Correlation coefficient *r*
EP	Stage 1	138.26	0.9964
	Stage 2	98.81	0.9998
PEI-rGO1.0/EP	Stage 1	149.26	0.9978
	Stage 2	93.12	0.9989
C_60_1.0/EP	Stage 1	162.33	0.9992
	Stage 2	111.59	0.9996
C_60_-PEI-rGO0.4/EP	Stage 1	155.80	0.9958
	Stage 2	101.93	0.9980
C_60_-PEI-rGO0.6/EP	Stage 1	192.72	0.9994
	Stage 2	108.13	0.9942
C_60_-PEI-rGO0.8/EP	Stage 1	216.66	0.9893
	Stage 2	100.40	0.9988
C_60_-PEI-rGO1.0/EP	Stage 1	224.31	0.9829
	Stage 2	105.5	0.9991

The result indicates that the initial thermal degradation that relate to the decomposition of the macromolecular chains becomes difficult with the addition of C_60_-PEI-rGO. It can be explained that C_60_ exhibits high efficiency on capturing radicals which were produced by the decomposition of the macromolecular chains, and it needs higher energy to keep decomposition which leads to the delay of decomposition. Meanwhile, no remarkable improvements on the value of E_a_ at 1st degradation stage by adding C_60_ alone are observed, which is due to the low specific interfacial area caused by poor dispersion. Obviously, the increased activation energy indicates that the combustion of epoxy resin is delayed and suppressed with the incorporation of C_60_-PEI-rGO. However, as the digital images and SEM images for the char shown in Additional file 1: Figure S2 and S3, respectively, the weight and microstructure of char are not obviously changed by incorporating C_60_-PEI-rGO, which is consistent with the results of cone calorimetry, indicating that the formation of char is not influenced by incorporating C_60_-PEI-rGO.

**Fig. 11:**
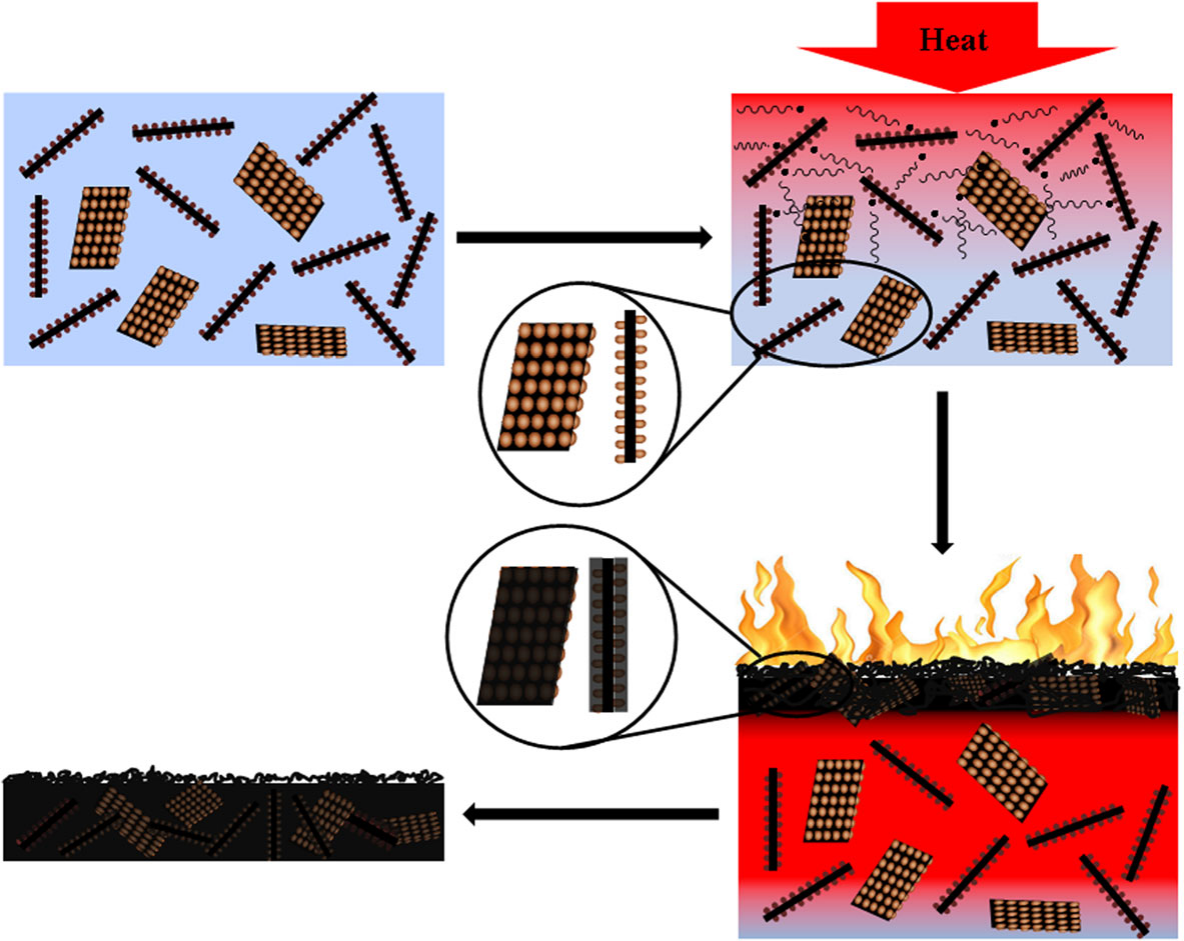
Schematic combustion processes of C_60_-PEI-rGO/EP nanocomposites

Based on the above discussion, a flame retardant mechanism is proposed as shown in Fig. 11. On the one hand, as discussed on the structure of EP and its nanocomposites, the amine groups in C_60_-PEI-rGO tend to shorten the distance among cross-linking points and increase the cross-linking density of the resultant nanocomposites which plays a positive role in improving the flame retardancy of EP. On the other hand, the synergy effect of C_60_ and graphene also plays the positive role in improving the flame retardancy of EP. Firstly, C_60_ aggregations with the size of 20 nm anchored evenly on the surface of PEI-rGO and the resultant large specific surface area can take them full use on trapping radicals and increases the activation energy of thermo-oxidative decomposition of EP chains. This effect delays the thermo-oxidative decomposition of the resultant nanocomposites, which reflects in some key index such as the time to ignition. Secondly, C_60_-PEI-rGO which possesses a layered structure acts as a physical barrier that hinders the transfer of volatile gases and heat. Finally, the combustion of nanocomposite is eased up and then the flame retardancy of C_60_-PEI-rGO/EP nanocomposites can be significantly improved.

### The Other Typical Properties of C_60_-PEI-rGO/EP Nanocomposites

Nowadays, there is a trend towards developing novel flame retardancy materials with simultaneously improved comprehensive properties rather than only attractive flame retardancy [4]. Therefore, it is necessary to evaluate other typical properties of C_60_-PEI-rGO/EP nanocomposites.

**Fig. 12:**
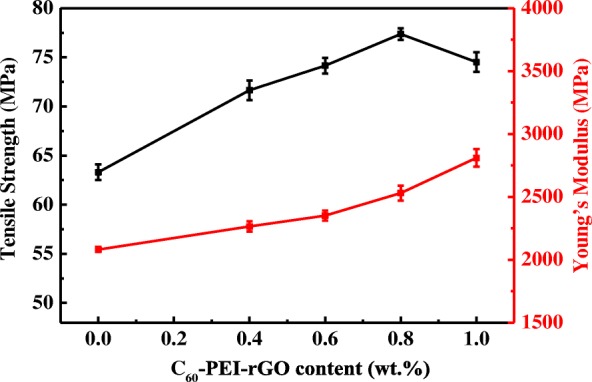
Tensile strength and Young’s modulus of EP resin and its nanocoposites

Figure 12 shows the tensile strength (T_s_), and Young’s modulus (Y_c_) of cured EP resin and resultant nanocomposites. It is attractive to find out that all C_60_-PEI-rGO/EP nanocomposites have higher values of tensile strength, and Young’s modulus than those of EP resin, demonstrating that C_60_-PEI-rGO/EP nanocomposites have the significantly improved tensile properties. Young’s modulus of C_60_-PEI-rGO1.0/EP reaches 2810 MPa, which is 1.35 times of EP (2081 MPa). Generally, the rough fractured surface is considered as a reflection on strong interfacial interaction between the polymer and graphene, which will lead to a high Young’s modulus [37, 39]. As shown in Fig. 6, it can be seen that PEI-rGO1.0/EP, C_60_-PEI-rGO0.6/EP and C_60_-PEI-rGO0.8/EP nanocomposites exhibit rougher fractured surfaces comparing with the neat EP. These results indicate that the stress can be effectively transferred between graphene layers and EP matrix through the interface phase, and the graphene layers fully exert their high stiff in nature and divert the course of crack propagation when exerting the load on the nanocomposites. The stronger force is needed to offset the absorbed energy by graphene layers and thus leads to the higher Young’s modulus of nanocomposites. However, the biggest incremental improvement of tensile strength occurs in C_60_-PEI-rGO0.8/EP, and the value up to 77.4 MPa, which is 1.22 times of cured EP. This can be explained that the high viscosity of C_60_-PEI-rGO1.0/EP leads to the difficulty on completely eliminating the organic solution which plays a negative role on the tensile strength of the resultant nanocomposite.

**Fig. 13:**
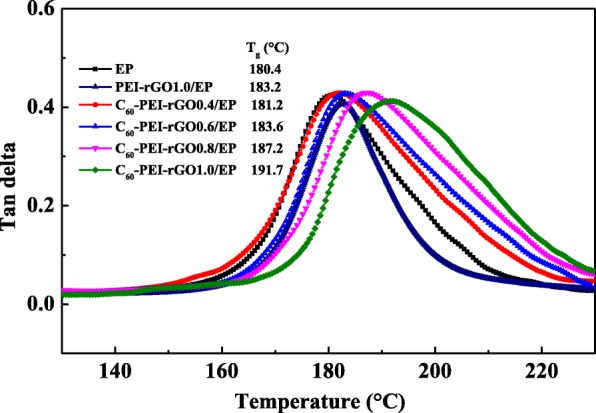
DMA curves of loss factor (tan delta) of cured EP resin and its nanocomposite

Figures 7 and 13 show DMA curves of cured EP and its nanocomposites, the storage modulus (*E*_s_) of cured EP significantly increase with the loading of C_60_-PEI-rGO, especially at lower temperature. The highest value of *E*_s_ (3125 MPa) occurs in C_60_-PEI-rGO1.0/EP, which is increased by 53.7% compared to that of neat EP (2039 MPa) at 30 °C. As the same trend as the *E*_s_, the *T*_g_ value of the C_60_-PEI-rGO/EP nanocomposite shifts towards higher temperature and the *T*_g_ value of C_60_-PEI-rGO1.0/EP is up to 191.7 °C which is an increment of 11.3 °C compared to that of neat EP. Meanwhile, PEI-rGO1.0/EP has slightly increased *T*_g_ and significantly increased *E*_s_ compared to neat EP, which accords with the results in other reports about functional graphene polymeric nanocomposites [39]. These result indicate that the functionalization of GO exhibits a positive effect on the properties of resultant nanocomposites. It is noted that the improvement of C_60_-PEI-rGO1.0/EP is more effective than that of PEI-rGO1.0/EP, which is attributed to physical interaction between C_60_ aggregations and EP matrix.

**Fig. 14:**
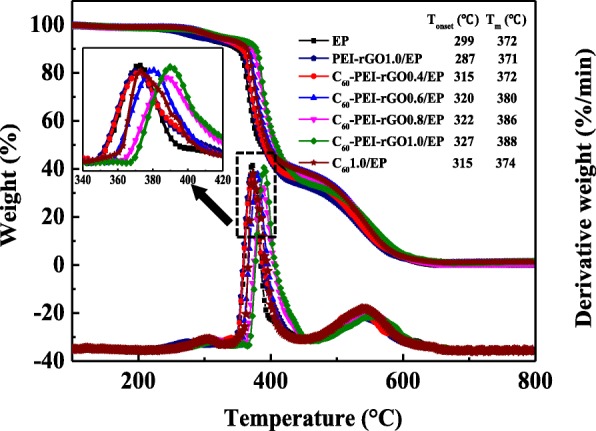
TG and DTG curves of cured EP resin and its nanocomposites in an air atmosphere

TG/DTG profiles for cured EP and its nanocomposites are shown in Fig. 14. The temperature (*T*_onset_) at 5 wt% of the weight loss of the sample and the temperature (*T*_max_) at maximum weight loss rate of samples are given. It can be seen that the thermal degradation process of neat EP has three stages, which mainly correspond to the vaporization of small molecules, the decomposition of the macromolecular chains, and the oxidation of char residue, respectively [20]. In case of PEI-rGO1.0/EP, the *T*_onset_ (287 °C) is lower than that of neat EP (299 °C), while *T*_m_ is not significantly changed, which could be due to the thermally unstable of PEI-rGO. For C_60_1.0/EP and C_60_-PEI-rGO/EP nanocomposites, *T*_onset_ and *T*_max_ are shifting to high temperature. Specially, C_60_-PEI-rGO1.0/EP exhibits the best thermal stability, the 28 °C increment of *T*_onset_ and 16 °C increment of *T*_max_ compared to that neat EP are observed. While for C_60_1.0/EP, the *T*_onset_ increases by 16 °C and has no significant change on *T*_m_, which could be due to the highly effective free radical-trapping effect of C_60_. However, the *T*_onset_ and *T*_m_ of C_60_1.0/EP are lower than those of C_60_-PEI-rGO/EP at equal content of nanofillers, which shows that C_60_-PEI-rGO is more effective than C_60_ or PEI-rGO alone in enhancing the thermal oxidation stability of EP. As described above, on the one hand, the layered structure of modified GO nanosheet increases the crosslinking densities of the resultant nanocomposites. Besides that, it creates a “Tortuous path” to form a gas barrier in degradation and provides a platform on which C_60_ could anchor evenly by chemical bond; the distribution of C_60_ in EP has improved. On the other hand, C_60_ acts as a radical trapping reagent during the process of degradation that delays the thermo-oxidative degradation of EP.

Layered nano-materials, such as graphene, clay, and layered double hydroxides, have been considered as potential multi-functional flame retardants. Comparing these nanomaterials, (i) the C_60_-PEI-rGO developed herein exhibits highly modified efficiency on flame retardancy of EP by combining multi-effects such as increase of crosslinking density, barrier effect of layered structure, and radical absorption of C_60_, and (ii) it endows modified resin with outstanding thermal stability and mechanical properties. Therefore, this work provides a new template to fabricate high flame retardant thermosetting resin with improved comprehensive properties.

## Conclusions

C_60_ was chemically anchored on the surface of PEI modified GO, and the resultant hybrid (C_60_-PEI-rGO) was successfully prepared. C_60_ aggregations with the size of ca. 20 nm are uniformly distributed on the surface of PEI-rGO, and C_60_-PEI-rGO exhibits a loose lamellar and amino-rich structure. The C_60_-PEI-rGO shows high flame retarding efficiency for EP. Specially, C_60_-PEI-rGO1.0/EP shows 40.0 and 15.6% reduction in the PHRR and THR compare to neat EP, respectively. More importantly, *t*_ign_ and times to PHRR of C_60_-PEI-rGO1.0/EP nanocomposite procrastinate for 21 s and 28 s compare to that of neat EP, respectively. This C_60_-PEI-rGO hybrid increases the crosslinking densities of the resultant nanocomposites and acts as a physical barrier that hinder the transfer of volatile gases and heat due to the layered structure; meanwhile, C_60_ aggregations are uniformly dispersed in EP resin by anchoring on the surface of PEI-rGO, acting as a radical trapping reagent which delays the thermo-oxidative degradation of the resultant nanocomposites. Moreover, it is noted that the C_60_-PEI-rGO not only is a high effective flame retardant but also is a potential nanofiller for fabricating high-performance thermosetting resins.

## Additional file

Additional file 1: Method (Preparation of Graphite Oxide, Preparation of PEI-rGO). **Table S1**. UL-94 results for cured EP and its nanocomposites. **Figure S1**. SEM image of fullerene (rapid removing ethanol). **Figure S2**. Digital photographs of char residues of cured EP (a), C_60_1.0/EP (b), PEI-rGO1.0/EP (c), C_60_-PEI-rGO0.4/EP (d), C_60_-PEI-rGO0.6/EP (e), C_60_-PEI-rGO0.8/EP (f) and C60-PEI-rGO1.0/EP (g) after cone test. **Figure S3**. SEM micrographs of residual chars for cured EP and m-C_60_-PEI-rGO1.0/EP. **Table S2**. Selected mechanical properties of cured EP and its nanocomposites. **Table S3**. Densities of cured EP and its nanocomposites. **Table S4**. The thermal conductivity of cured EP and its nanocomposites. (DOCX 2316 kb)

## Abbreviations

AFM: Atomic force microscope
C_60_: Fullerene
DETDA: Diethyltoluenediamine
DGEBA: Diglycidyl ether of bisphenol A
DMA: Dynamic mechanical analysis
DMSO: Dimethyl sulfoxide
EP: Epoxy
FTIR: Fourier transform infrared spectrometer
GO: Graphene oxide
LOI: Limiting oxygen index
PEI: Branched polyethlyamine
PHRR: Peak heat release rate
rGO: Reduced graphene oxide
SEM: Scanning electron microscope
TEM: Transmission electron microscopy
TGA: Thermogravimetric analyses
THR: Total heat release
TSR: Total smoke release
