# Supplementary material for: Fabrication of Fullerene Anchored Reduced Graphene Oxide Hybrids and Their Synergistic Reinforcement on the Flame Retardancy of Epoxy Resin
Source: Nanoscale Res Lett. 2018 Nov 3;13:351. doi: 10.1186/s11671-018-2678-z (PMC6215538; doi:10.1186/s11671-018-2678-z)
Supplement: Supplementary file 1 — Method (Preparation of Graphite Oxide, Preparation of PEI-rGO). Table S1. UL-94 results for cured EP and its nanocomposites. Figure S1. SEM image of fullerene (rapid removing ethanol). Figure S2. Digital photographs of char residues of cured EP (a), C601.0/EP (b), PEI-rGO1.0/EP (c), C60-PEI-rGO0.4/EP (d), C60-PEI-rGO0.6/EP (e), C60-PEI-rGO0.8/EP (f) and C60-PEI-rGO1.0/EP (g) after cone test. Figure S3. SEM micrographs of residual chars for cured EP and m-C60-PEI-rGO1.0/EP. Table S2. Selected mechanical properties of cured EP and its nanocomposites. Table S3. Densities of cured EP and its nanocomposites. Table S4. The thermal conductivity of cured EP and its nanocomposites. (DOCX 2316 kb) [file 11671_2018_2678_MOESM1_ESM.docx]

**Supporting Information**

**Fabrication of fullerene anchored reduced graphene oxide hybrids and their synergistic reinforcement on the flame retardancy of epoxy resin**

Rui Wang^1, 2^, Lixin. Wu^1^*, Dongxian Zhuo^3^*, Zhengzhou Wang^4^ and Tsung Yen Tsai^5^

^1^Fujian Institute of Research on the Structure of Matter, Chinese Academy of Sciences, Fuzhou 350000, P. R. China. E-mail: wangrui@fjirsm.ac.cn; [lxwu@fjirsm.ac.cn](mailto:lxwu@fjirsm.ac.cn)

^2^University of the Chinese Academy of Sciences, Beijing 100049, P. R. China

^3^Quanzhou Normal University, Quanzhou 362000, P. R. China. E-mail: [dxzhuo@qztc.edu.cn](mailto:dxzhuo@qztc.edu.cn)

^4^School of Materials Science and Engineering, Tongji University, Shanghai 201804, P. R. China. E-mail: 172431330@qq.com

^5^Department of Chemistry, Center for Nanotechnology, R&D Center for Membrane Technology, Chung Yuan Christian University, Chungli 32023, Taiwan. 1466811684@qq.com

* Correspondence: [lxwu@fjirsm.ac.cn](mailto:lxwu@fjirsm.ac.cn); [dxzhuo@qztc.edu.cn](mailto:dxzhuo@qztc.edu.cn)

**Methods**

**Preparation of Graphite Oxide**

Graphite oxide was prepared using a modified Hummer’s method from graphite powders. Typically, 3 g graphite, 1.5 g NaNO_3_, and 144 mL of 98 wt.% H_2_SO_4_ were added to a three-necked bottle in an ice bath with vigorous stirring. Subsequently, 9 g KMnO_4_ was slowly added, and the mixture was reacted at 15 ^o^C for 1h and 50 °C for 30 min; then 144 mL distilled water was slowly added to the mixture which was below 70 ^o^C. Afterward, temperature raised to 98 ^o^C and maintained for 15 min, and then the mixture was poured into 600 ml of 10 wt.% H_2_O_2_ solution. Finally, a yellow powder was obtained after repeatedly washing with distilled water and completely drying at 50 ^o^C in a vacuum oven, and named as GO.

**Preparation of PEI-rGO**

50 mg of graphite oxide was dispersed in 500 mL of distilled water by sonication for 30 min. After that, 200 mg of PEI was added and heated at 60 ^o^C for 12 h with stirring after ultrasonic for 30 min at room temperature. Subsequently, the suspension of product was washed 5 times with ethanol followed by drying it at 60 ^o^C under vacuum overnight and the product was designated as PEI-rGO.

Table S1 UL-94 results for cured EP and its nanocomposites

| Samples  (3.2mm) | *t*_1_ (s) | *t*_2_ (s) | Dripping | Rating |
| --- | --- | --- | --- | --- |
| EP | > 30 | / | Yes | No rating |
| PEI-rGO1.0/EP | 49 | 58 | Yes | No rating |
| C_60_1.0/EP | > 30 | / | Yes | No rating |
| C_60_-PEI-rGO0.4/EP | 52 | 65 | Yes | No rating |
| C_60_-PEI-rGO0.6/EP | 48 | 52 | Yes | No rating |
| C_60_-PEI-rGO0.8/EP | 37 | 42 | No | No rating |
| C_60_-PEI-rGO1.0/EP | 35 | 46 | No | No rating |


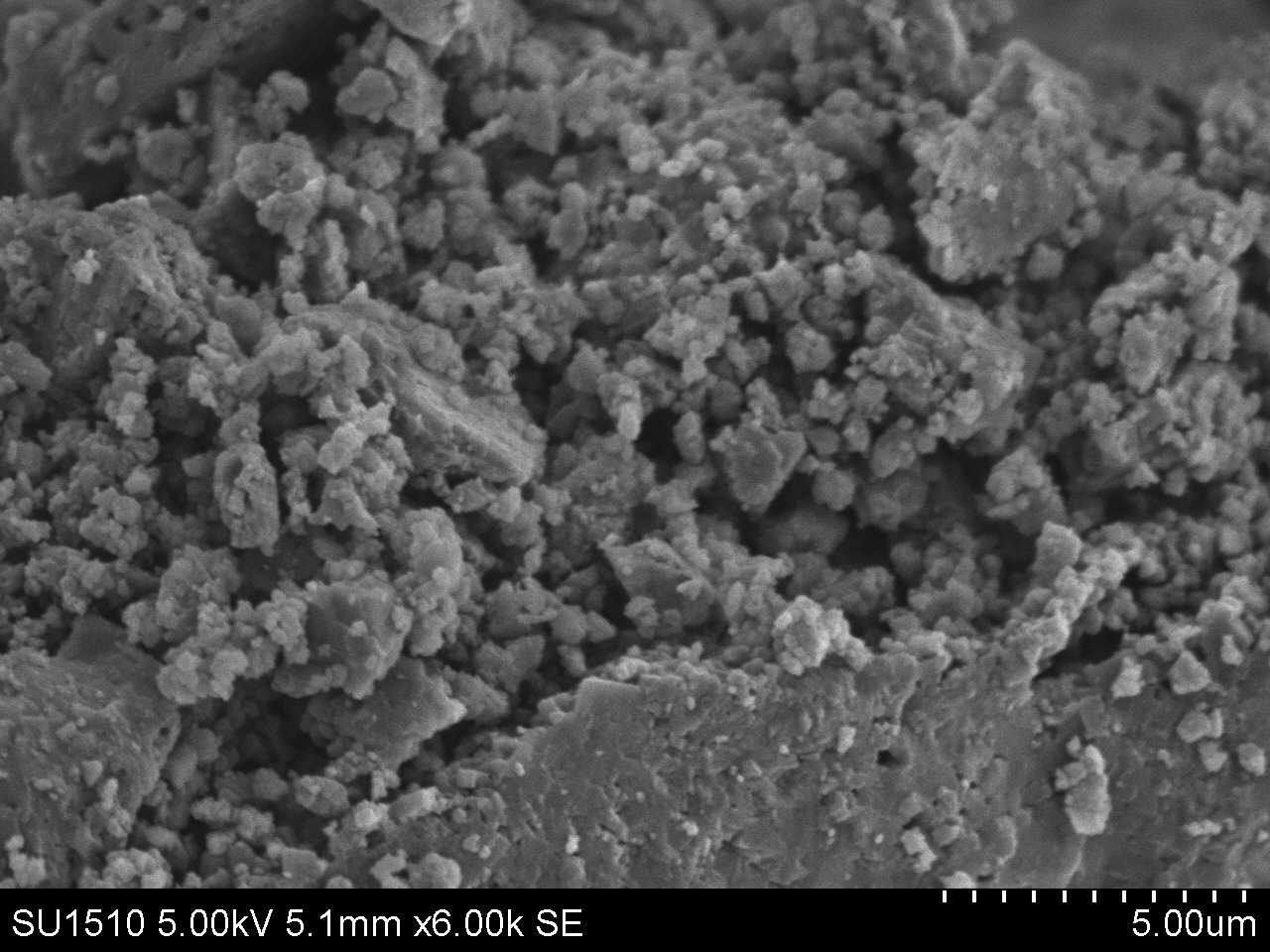


Fig. S1 SEM image of fullerene (rapid removing ethanol)


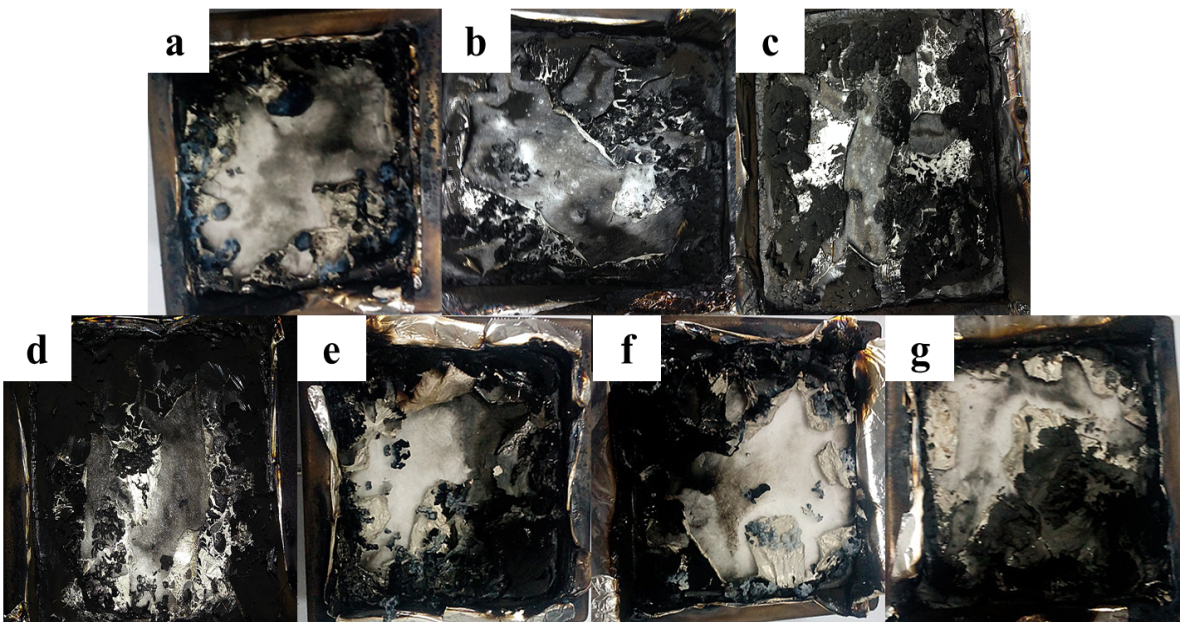


Fig.S2 Digital photographs of char residues of cured EP (a), C_60_1.0/EP (b

), PEI-rGO1.0/EP (c), C_60_-PEI-rGO0.4/EP (d), C_60_-PEI-rGO0.6/EP (e), C_60_-PEI-rGO0.8/EP (f) and C_60_-PEI-rGO1.0/EP (g) after cone test.


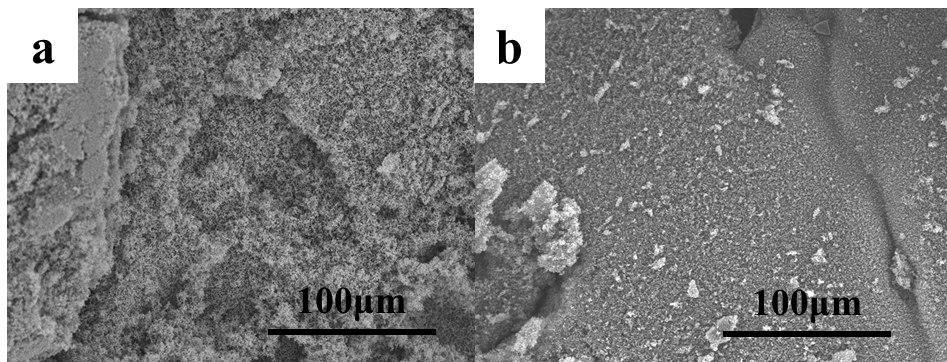


Fig. S3 SEM micrographs of residual chars for cured EP and m-

C_60_-PEI-rGO1.0/EP

Table S2 Selected mechanical properties of cured EP and its nanocomposites

| Samples | Tensile Strength | Young’s Modulus | Elongation at  break | Impact Strength |
| --- | --- | --- | --- | --- |
| EP | 63.2±1.1 | 2081±32 | 4.8±0.2 | 24.5±9.6 |
| C_60_-PEI-rGO0.4/EP | 71.6±1.2 | 2265±54 | 4.1±0.5 | 29.8±12.7 |
| C_60_-PEI-rGO0.6/EP | 74.0±0.9 | 2347±60 | 3.8±0.6 | 27.6±11.5 |
| C_60_-PEI-rGO0.8/EP | 77.4±0.8 | 2530±73 | 3.7±0.5 | 31.2±14.5 |
| C_60_-PEI-rGO1.0/EP | 74.4±1.4 | 2810±86 | 3.3±0.7 | 27.3±18.5 |

Table S3 Densities of cured EP and its nanocomposites

| Samples | Density (g/cm^3^) |
| --- | --- |
| EP | 1.164 |
| C_60_-PEI-rGO0.4/EP | 1.166 |
| C_60_-PEI-rGO0.6/EP | 1.171 |
| C_60_-PEI-rGO0.8/EP | 1.168 |
| C_60_-PEI-rGO1.0/EP | 1.159 |

Table S4 The thermal conductivity of cured EP and its nanocomposites

| Samples | Thermal conductivity (W/mk) |
| --- | --- |
| EP | 0.21 |
| C_60_-PEI-rGO0.4/EP | 0.20 |
| C_60_-PEI-rGO0.6/EP | 0.24 |
| C_60_-PEI-rGO0.8/EP | 0.23 |
| C_60_-PEI-rGO1.0/EP | 0.19 |
